# Adapting Bodies to Infrastructures

**DOI:** 10.1007/s10746-021-09578-3

**Published:** 2021-04-09

**Authors:** Larissa Schindler

**Affiliations:** Faculty of Humanities and Social Sciences, University of Bayreuth, Soziologie / GW II, 95440 Bayreuth, Germany

**Keywords:** Materiality, Ethnography, Passengering, Practice theory, Air travel, Infrastructure, Sociology of the body, Body-object-interrelations, Mobilities

## Abstract

The material interrelations between bodies and objects are a wide, worthwhile and absorbing field, which has not been sufficiently examined yet. Focusing on such interrelations within air travel, this article contributes to the exploration of this field. It delineates that such interrelations do not simply happen, but that they have to be accomplished continuously by different participants with a certain risk to fail at many points. Within mobilities, such processes of interrelating occur under the specific circumstances of a moving vehicle. Based on empirical data from an ethnographic study on air travel, the paper is concerned with the ongoing dynamics of body-object-interrelations. Based on this analysis, it suggests emphasizing the continuous modification that material assemblages built of people and objects undergo even when “only” staying immobile during a flight. Passengers do not simply enter and exit a vehicle staying identical with themselves. Rather, they are continuously adapting to a material infrastructure that shapes and adjusts their corporeal needs and capacities, including their senses.

## Introduction

The materiality of the social has been widely discussed in the last four decades. Numerous studies have shown that we cannot fully understand social processes if we ignore the contribution of objects (e.g., Knorr Cetina 2000; Latour 1996; Law 1999) and infrastructure (e.g., Parks 2015; Star 1999), since they considerably contribute to the course of social processes. We live in a built environment of houses and streets; we use vehicles to be mobile and digital media to communicate. These things not only support our everyday lives but also shape and restrict our doings and thus participate in constantly (re)building social order. At the same time, in a separate strand of discussion, (human) bodies have gained the attention of social and cultural sciences (see Shilling 2005, 2007), since they appear to be shaped by social processes as much as they contribute to shaping them. Not always do persons’ intentions correlate with their body’s activities, which may interfere with, support, or stay neutral to these intentions. Accomplishing practices, however, deeply depends on our bodies. This is evident in physical practices like dancing or sports, yet it also applies to prima facie cognitive work, such as handling a computer, which not only requires a body in front of the display operating the keyboard but consists of many different embodied activities (see Schmidt 2008).

As the example of the embodied work of using a computer adumbrates, the materiality of objects and (human) bodies may currently be discussed in separate theoretical strands; empirically, however, they are often connected to each other. Perhaps the most prominent case of such material connections is medical procedures that are based on a profound embodied knowledge of doctors, for instance when operating (Schubert 2011a), as well as on a technical extension of doctor’s senses (e.g., Lachmund 1999; Schubert 2011b). On the patients’ part, medical procedures require embodied interactions with different technologies and artifacts in order to establish a technical extension of the medical gaze or (in the case of prosthesis and implants) a technical substitute for organs. Since medical procedures have a strong impact on one’s own perception of sovereignty, they are often discussed in terms of morality (e.g., Verbeek 2008) or agency (e.g., de Boer and Slatman 2018). Less jarring, yet also theoretically fruitful, are the numerous cases of body-object-connections that we find in the wide field of physical movement, be it in mobilities or in sports. From walking (with shoes) to ball games, skating, cycling, or driving to railway or air travel: the interrelations of bodies and objects are obviously crucial for accomplishing movement and have been addressed in a number of case studies (e.g., Dant 2004; Hirschauer 2005; Winance 2006). Finally, modern everyday lives give numerous examples of such connections, since the sociability of our bodies depends on clothes, food, and countless other objects like glasses, phones, watches, documents, tickets, and the like. These objects serve as extensions or augmentations of our bodies, as substitutes for embodied activities, or as media of communication, of social performance, and/or social distinction. In short: The field of body-object interrelations is a wide, differentiated, and complex one. Against this background, it is not surprising that (as of yet) there is no theoretical conception suitable for all such cases. However, even within the range of empirical studies, there are still numerous gaps.

Focusing on processes of adapting bodies to the infrastructure of an airplane, I address one of these gaps. Based on empirical data from an ethnographic study on air travel, I am concerned with practices of “passenger-ing” (e.g., Ashmore 2013; Bissell et al. 2011; Laurier et al. 2008). In this analysis, the ongoing dynamics of body-object interrelations become evident, since passengers are continuously engaged in adapting their bodies to the given infrastructure of the airplane that forces them to move as little as possible. While modern societies are usually emphatic about “mobile bodies” (Imrie 2000), within many vehicles, bodies are widely restricted to micro-movement, i.e., activities like reading, eating, or sleeping. Thus, air travel is a fruitful case for understanding how the immobilization of bodies requires an ongoing practice of interconnecting bodies and objects with the given infrastructure.

Thus, in what follows, I take up the field of mobilities to contribute to the ongoing discussion on the materiality of the social by focusing on the interrelations between bodies, objects, and infrastructure. My argument is developed as follows: Firstly, I discuss in detail the theoretical starting points of the proposed analysis. Secondly, I briefly sketch the methodological assumptions of the empirical data used. Thirdly, I start the empirical analysis by focusing on the passengers entering the plane. At this point, they are preparing to become part of the plane assemblage, which widely prohibits them from moving within the plane, i.e., they widely give up their bodies’ mobility. Fourthly, I handle practices of accommodating to the infrastructure of the plane, particularly to seats. Here, objects (provided by the airline or brought along by the passenger) play a crucial role as “adapters”. However, this is not done once forever. Thus, I am fifthly concerned with the ongoing (and fairly demanding) accomplishment of adapting bodies to the given infrastructure, i.e., of staying nearly immobile for the entire flight. Sixth, (mostly covert) conflicts with other passengers delineate the complexities of adapting passengers and their bodies to the infrastructure. Seventh, practices of detaching from the vehicle are addressed, which en passant reflect these complexities. My analysis finds that during air travel, passengers, their bodies and objects take part in different assemblages. Thus, they do not remain self-identical throughout the entire travel, but rather they continuously adapt to the material infrastructure that, in change, shapes and adjusts their corporeal needs and capacities.

Thereby, my contribution also touches upon a more macro-sociological topic within mobilities research: While it is certainly true that the automobile has shaped modern societies in profound ways (e.g., Dant 2004; Sheller and Urry 2002), the experience of hetero-mobility (Hirschauer 2005: 48) has also had a deep impact on modern living. Our everyday lives are characterized by using escalators, moving pavements, or elevators and by traveling as passengers in cars, busses, trains, or airplanes. Thus, in addition to practices of driving, practices of “passengering” also deserve analytical attention.

### Theoretical Starting Points: Bodies and Objects in Mobilities

As I have already mentioned in the introduction, currently, the crucial contributions of bodies and objects to the social are usually discussed in different strands. However, as in many other similar cases, there are exceptions in this general trend. For research on the interrelations of bodies and objects within mobilities, the following contributions offer important theoretical starting points:

Strikingly, there are promising starting points in the work of some classics: One of these is Mauss’ famous speech “Body Techniques” from the 1930s (English translation in 1979). Often quoted, he declares the body as man’s first technical object (Mauss 1979: 75) and thus suggests that the human body (as an object) can be analytically separated from humans (and their embodied activities). Schützpelz (2010: 111) convincingly concludes from this approach that the strict separation of (human) bodies and technique is implausible. Based on his reading of Mauss, he suggests drawing a continuum from (1) embodied skills (like running or sleeping) to (2) skills of using tools to (3) replacing human activities with objects (e.g., traps). Thus, in this conception embodied and technically augmented activities are closely related, a suggestion that holds high plausibility in the fields of mobilities, as I have delineated in the introduction.

Decades after Mauss, Goffman (1971) suggests another approach of body-object interrelations in his famous book “Relations in Public,” investigating different forms of interaction in which the modern individual can engage. One of these forms is taking part in urban traffic as a “vehicular unit,” (Goffman 1971: 26–40) a term that immediately associates the individual with technology. Actually, in Goffman’s account, a car can be a vehicular unit as well as a pedestrian. He defines it as “a shell of some kind controlled (usually from within) by a human pilot or navigator” (Goffman 1971: 26) that participates in road traffic. Thus, in his approach, there is little importance for the question whether a vehicular unit depends on embodied skills or on the technical replacement of human movement. Rather, the analytical interest lies in the question how these different units interact with each other.

Both approaches thus circumvent the current separation between bodies and objects as participants of social processes. However, in both approaches we find a certain bias towards functionalist descriptions of such processes. Pauses, interruptions, difficulties, confusions, and the like are not included in the analysis. Yet, both strands of the current discussion, i.e., body- and object-related analysis, emphasize that bodies or objects usually remain unnoticed until at some point they do not work fluently anymore. Usually, such moments are considered to be of high analytical value, since they make the social activities of bodies as well as of objects easier to detect (e.g., Winance 2006: 54).

In this vein, currently, different contributions focus on the complex dynamics of body-object interrelations within mobilities. Their main points are that (1) in modern societies human bodies are hardly ever “unprostheticised by various technologies” (Spinney 2006: 715) and (2) that humans and (their) vehicles are not separated units, but only together form an assemblage (e.g., Dant 2004) or a common materiality (Winance 2006) that brings about specific types of action and prevents others. In this way, these assemblages produce specific possibilities of taking part in social processes. Among these studies, we find a certain preference for “extended bodies” (Winance 2006: 61), such as the “driver-car” (Dant 2004), the “human–machine with wheels” (Alkemeyer 2006) of motorbikes, or the “body-in-the-wheelchair-of-the-person” (Winance 2006: 58). In other words: most of these studies apply an analytical angle that focusses on the materialities of piloting a vehicular unit. Convincingly, they delineate that these assemblages produce specific awarenesses for the environment (e.g., Allen-Collinson 2008; Spinney 2006) as well as requiring particular forms of practical knowledge (e.g., Alkemeyer 2006; Spinney 2006). Also, as Spinney (2006: 717) argues, bicycle and rider develop “in conjunction with one another through practical use”. In this perspective, not only micro but also macro-sociological phenomena are addressed, as for instance the impact of the automobile on modern societies (Dant 2004: 62).

In what follows, I build on the manifold insights these contributions give. However, I focus on a phenomenon that (so far) has had less attention: the body-object interrelations of passengers, i.e., the common materialities of widely immobilized bodies in modern means of mass transport, bodies that are (technically and socially) incorporated into a vehicle. For passengers, such vehicles are neither a tool (since they do not drive/pilot them) nor do they simply replace human bodies’ activities (like a trap); they are means of transport that incorporate human bodies as (mainly still) passengers. From this perspective, further peculiarities of modern body-object interrelations come into view, particularly the continuous work of adapting one’s body to the infrastructure of the vehicle without having any means to influence the course of the ride. Passengers cannot stop the vehicle, and in many cases, they cannot even leave their seats without facing considerable inconveniences. The airplane is a fruitful case for exploring such processes, since a flight takes relatively long and, at the same time, passengers are intensively immobilized. One could say that being an air passenger is mostly accomplished by reducing one’s activities to mostly immotile ones.

Thus, I follow the lead of a (small) number of publications that have brought up the topic of passengers and “passengering,” indicating that within a vehicle a number of different activities (in addition to driving or piloting it) take place, which are worth being studied in detail. In this line, Laurier and colleagues (2008) have elaborated on conversations and arrangements of visibilities in cars, suggesting that the space of the car forms a “translation and displacement” of other, exterior spaces, like offices and homes (Laurier et al. 2008: 19). Although within media of mass transport like trains or ships, conversations are mostly held with acquainted fellow travelers, Bissell (2010: 271) points to “affective registers of communication” and their significant impact on the (individual and collective) experience of a journey. In this study of affective atmospheres in trains (Bissell 2010), he highlights the peculiar sociality of train carriages that is based on hybrid constellations of bodies and matter. Similarly, Ashmore (2013) delineates the affective atmospheres of an inter-war ocean-liner with a focus on characteristics of long-distance maritime voyages. Although many of these contributions consider “passengering” to be a multi-faceted skill (e.g., Vannini 2011: 1031) and (within their interest in affective atmospheres) reflect upon objectual and embodied dimensions of the social, to my knowledge there is no contribution that particularly focuses on the materiality of body-object-relations as a basic component of social practices. In this regard, my paper also contributes to the growing field of studies on “passengering”.

### Methodology

The empirical data for the following analysis are drawn from an ethnographic study on air travel which I conducted between 2016 and 2020.^1^ It focussed on the embodied dimensions of technically augmented mobility. As the research period indicates, the empirical research was designed and carried out before the coronavirus outbreak. Since then, air travel (like most other segments of social life) has seen profound changes: an immense decline in the number of flights and—on the remaining flights—different measures of social distancing as an attempt to prevent (at least avoidable) infections. For the most part, the interrelations between bodies and objects within air travel are only affected by these changes on the surface. Further controls, obligations to wear facemasks, and other related measures, certainly make a flight even more stressful and tiring, yet they do not replace or remove all the other corporeal challenges which flights already presented before.

Theoretically, the research project followed a practice-oriented approach (e.g., Reckwitz 2002; Schatzki et al. 2000), which emphasizes the role of (embodied and objectual) materialities and of temporalities for social dynamics. From the wide range of approaches that has inspired practice theories, my analytical perspective was in particular influenced by studies in ethnomethodology (as coined by Garfinkel 1967) and by research on the interaction order (e.g., Goffman 1963, 1983). Thus, situational dynamics of accomplishing practices are at the core of my considerations, albeit with a notable focus on material dimensions of the social (as i.a. proposed by Hirschauer 2004, 2005; Knorr Cetina 2000, 2009).

Due to this focus on material dimensions, I deployed an ethnographic research design that particularly drew on the researcher’s social, embodied, and sensory skills in order to reconstruct social processes including their material (and often silent) dimensions (see Hirschauer 2006; Kalthoff 2013). Following the ethnographic approach (e.g., Breidenstein et al. 2013), the methodology was based on context-sensitive methods as well as on the combination of different data. By the end of the research project, the corpus consisted of field notes documenting 49 short-, medium- and long-haul flights (including photos and some short videos), 24 qualitative interviews with mainly passengers, but also flight attendants and pilots, and 21 “logbooks” (see below) written by passengers.

Like most fields of investigation, airplanes provide a particular setting for applying and combining different methods, which themselves have particular advantages and disadvantages. Participant observation as a mobile method provides the researcher with deep insights into tacit and embodied experiences of air traveling. At the same time, such observations suffer from the narrow view in the plane and from their limitation to present events. Interviews, on the other hand, widen the perspective of the study considerably, since they include the views of other passengers and the crew. Also, they can provide background knowledge and inform about events before and after a flight. At the same time, interviews are restricted by informants’ ability of reporting (see Cuff and Francis 1978; Hester and Francis 1994) and they are usually conducted days or even weeks after the flights in question.

Bearing this gap in mind, I added a rather sparsely used method to the research design: I asked passengers to write about their experiences during or shortly after air travel. In sum, I have received 21 such “logbooks” on short- and long-distance flights.^2^ Like interviews, these are “invited stories” (Cuff and Francis 1978) as they would not have been produced without my request. However, they are less influenced by the researcher due to her absence from the writing process. Also, as logbooks are closer in time to the actual flight than interviews, they can be characterized as a “mobile method” (Büscher et al. 2010). As such, they add further perspectives to the field notes of my participant observations, which is crucial particularly for all areas with limited access for participant observation as e.g., security check (for a detailed discussion on these limitations: Pütz 2012).

Data was analyzed according to the principles of qualitative research, which were paradigmatically formulated in Grounded Theory (e.g., Glaser and Strauss 1967). Iterative gathering and analyzing of data leads to an analytical focus and thus ensures continuous quality control. The combination of different types of data is particularly influenced by Kalthoff’s (2010) considerations on triangulation. Since different methods generate different phenomenal domains, he suggests using the emerging differences prolifically.

### Interconnecting Body and Plane

Air travel does not start at the door of the plane. Rather, it usually begins weeks, sometimes even months earlier, when a ticket is booked. A lot of information has to be procured, documents have to be prepared, and luggage has to be packed according to transport guidelines and with a certain knowledge about necessities on board. In some cases, the traveler also needs to sort out vaccinations or other medication in advance. On the day of travel, a mobile assemblage^3^ fit to travel (as a vehicular unit) has to be built of the body and the travel objects—it leaves the house, reaches the airport and passes through it to the gate. On this route, different controls take place, in which the traveling assemblage has to disassemble and reassemble its components (see Schindler 2015). Sometimes, the body needs small transformations as well, like food, drinks, chewing gum, medication or changing clothes for the flight. In this way, “doing being a traveler” also consists in (accountably) adapting one’s body (and baggage) for the chosen medium of transport, its particular materialities, and the spaces it travels through.

At the door of the plane however, travelers become air passengers in a narrow sense, i.e., they become part of the airplane assemblage, which comprises bodies and objects within a flying machine (navigated through the skies by pilots in a closed cockpit). Therefore, passengers socially and materially have to adapt to the plane’s infrastructure, i.e., their bodies and objects must become closely interconnected with the vehicle. Within airliners, travelers usually meet the following, peculiar conditions: For safety reasons, passenger’s bodies and objects have to be part of an assemblage that prevents them from converting into projectiles in case of severe turbulences. For economic reasons, they should occupy as little space in the airplane as possible. For humanistic reasons however, a clear distinction between humans and non-humans has to be maintained, inter alia by conceding passengers (at least some) more space than the material body alone would need and allowing them to carry (at least some) personal items with them. Yet, it remains open to discussion whether this suffices to create “territories of the self” (Goffman 1971: 51–86) as we are used to outside. In the airplane, the material and social efforts of human physical travel thus become particularly evident.

These conditions lead to particular dynamics that are, in a way, already anticipated at the entrance of the plane, where passengers usually have to wait in a queue before they can enter.^4^ This queue results from the typical design of airliners with their tight seat rows and narrow aisles. Finding one’s seat, storing hand luggage, and accommodating into the seat is a sluggish procedure, especially on a full plane. It slows down the queue in the aisle and thus extends the one at the entrance. In a way, this waiting anticipates the immobile time passengers are going to spend during the flight. The following excerpt from my field notes highlights the troubles that can emerge at this stage, while a second one reports unusual ease:

Right after the queue in front of the airplane comes the queue inside. The aisle is full of people and the overhead bins are full—there is no place for my suitcase. I ask my seat neighbors to put my jacket and my bag on my (window) seat, looking for a solution for the suitcase. A lady with a suitcase is coming along the aisle towards me, that is from the rear to the front. I look at her and at her suitcase and then I say “funny problem”—she doesn’t react. (…) I ask my seat neighbors in English to get up to let me put the suitcase to my seat. They squeeze out of the row—the lady with the suitcase doesn’t move. A little annoyed, I tell her that I will put my suitcase into the row in order to let her pass. (Field notes, short-haul, November 2013)

This time, entering the plane is extraordinarily easy. There is nobody behind me, as I was the last one to come in. Also, those who are seated in the front of the plane board from the front, while I have come in from the rear. I put my jacket into the overhead bin and sit down in my aisle seat: Nobody has to get up for me, I don’t have to squeeze into the seat row. (Field notes, short-haul, November 2016)

Moving inside an airplane to one’s seat can be as easy as in the second excerpt or much more complicated. However, due to the common design of airliners, it differs considerably from the conditions of moving we are used to outside.^5^ Most noticeable, the aisle of an airliner is just wide enough for one person. Moving in a crowd, as pedestrians in many public places, or in more than one lane, as in urban traffic, is not possible. Additionally, the way to one’s seat is full of obstacles: the seat rows on the side, other passengers and their luggage. Being in a semi-public place however, everybody cooperates in accomplishing “civil inattention” (Goffman 1963: 83–88) and—as a crucial element of it—passengers cooperate in avoiding physical contact with other humans and (preferably) with others’ personal items. As described in the first excerpt, this can be complicated, carrying some potential for conflict. Due to the severely limited space, the situation often is socially fragile, many little problems between passengers have to be solved: by arranging material objects and bodies in the cabin, and sometimes by giving up civil inattention for a short conversation, as in the first excerpt.

Yet, the incoming passengers are still, in Goffman’s (1971: 26–40) terms, vehicular units of their own, pedestrians moving into a motionless plane, albeit this has to be accomplished under relatively dense circumstances.^6^ As passengers arrive at their seats however, they begin to become part of a bigger vehicular unit that will soon roll across the runway to take off into the skies. Storing hand luggage and taking one’s seat is the first step towards this transformation, fastening the seatbelt the second. At this point, similar to a ride in an elevator (Hirschauer 2005: 51), passengers have to change from navigating their own movements into a mode of stillness within an externally controlled flight. They have to accept that someone else, here: pilots in the cockpit (supported by technical tools and the information of flight controllers) navigate their ride, i.e., passengers take place in a “heteromobile” (Hirschauer 2005: 48). Compared to an elevator, the period of stillness lasts long, and compared to a train, opportunities to move are rare. Awaiting arrival, passengers spend most of the ride seated (in a flying cabin), constrained by the tight seat rows and held in place by a seatbelt.

With this transformation towards being part of the vehicular unit of the plane, specific challenges for “body-boundary-work” (Boll and Müller 2020) emerge: Accomplishing a stable interconnection with the vehicle by remaining seated impairs the experience of having a “mobile body” (Imrie 2000), most travelers are used to otherwise. At times, e.g., during turbulences or upon touch-down (see below), the interconnection even becomes so dense that passengers feel a kind of “common materiality” (Winance 2006) with the vehicle and their fellow travelers.

Materially, “doing being an air passenger” primarily consists in staying seated throughout the entire ride, and thus remaining in a rather stable, embodied interconnection with the vehicle. However, despite being a pivotal element of air travel, the otherwise mundane practice of sitting meets particular conditions in the plane and requires respective adaptations from the passengers. These are elaborated in the following sections.

### Accommodating: Objects as “Adapters”

In everyday life, sitting in chairs is often taken as a natural state. Historical and anthropological studies, however, find different cultures of sitting that vary considerably. Mauss (1979: 81) proposes distinguishing squatting from sitting mankind. Cranz (2000: 25–65) emphasizes that the high impact of chairs for western cultures (and the varieties in design) only has a short history of a few centuries. However, although sitting in chairs has become a common practice here, it has not yet received much analytical attention. As Bissell (2008: 1702) states (in regard of research on mobilities): “For all the attention that walking has and continues to receive, (…) it is the case that the chair is frequently the site of the practice of everyday life”. Indeed, we not only sit down to relax, but, while being seated, we also accomplish a whole range of activities like eating, reading, writing, meeting people in cafés or restaurants, many forms of work, attending plays or classical concerts, and: traveling by (many forms of) vehicles.

From a cultural theorist’s perspective, the practice of sitting in a chair or a seat can thus be considered a well-established technique of the body in western cultures. It is one of the basic techniques which children learn in the first year of their lives and are forced to “professionalize” at school. In this course, the practical knowledge of sitting down and remaining seated while accomplishing different other activities becomes nearly completely tacit. Most adults associate sitting with comfort, also because the design of chairs and seats is part of a continuous development towards ergonomics and comfort. Yet this feeling derives not only from the object but also from their embodied techniques of sitting. Bissell (2008: 1704) emphasizes that the whole body, i.e., the back, the neck, the arms and legs, are enlisted in producing a comfortable sensation while sitting. “(T)he seated body is constantly refiguring and becoming refigured through the cultivation of a sensibility of comfort,” he (2008: 1704) argues. Constantly adapting to the chair is an important aspect of the practical knowledge necessary to remain seated for a long while.

Sitting in an airplane, however, happens under particular conditions: Firstly, for the whole duration of the flight, there is hardly any room to move for the individual due to the given setting, and at the same time, due to explicit rules, possibilities to stand up are rare. This alone might presumably suffice to convert sitting into a sort of corporeal challenge. Secondly, however, the whole vehicle is constantly moving through the skies, including possible turbulences. Thus, being seated here loses part of the stillness that it has on firm ground. Thirdly, due to this movement and its possible dangers, passengers are obliged to use a security belt that further fixes them to the seat. Fourth, although seats are technically designed to be convenient, the design heavily draws on the physical norms of an average body that many bodies do not fit into.^7^ Fifth, passengers are situated among other passengers, i.e., they are constantly confronted with (mostly unknown) fellow travelers, their habits, bodies, and objects. Thus, sitting in a plane presents a number of corporeal challenges that result from the technicalities of flying human bodies through the skies.

Facing these challenges of sitting (sometimes over hours) in an airplane, passengers not only rely on their practical knowledge from outside but tend to develop strategies for its peculiarities. This includes strategic knowledge about how to spend the given time on board and about what to bring along in order to facilitate the sitting time (often related to the physical characteristics of one’s own body and one’s own habits). In this regard, some travelers reported on detailed lists of objects they carry with them only for the flight. These include quite different items ranging from expectable ones like inflatable cushions or magazines to more specific ones like a silk scarf to protect against the possibly cold air. Most of these items are used right at the outset of a flight in order to actively accommodate to the setting. The following excerpt from a logbook reports on such an activity:

But I don’t want to miss the take-off. So I get myself situated (get out book and iPad, put on socks, unpack blanket, stow headphones in the seat pouch, put on silk scarf, place cardigan on lap) while keeping an eye on the safety instructions. They’re done in a funny way to keep you waiting for the gags and make you pay a bit more attention. We taxi to the runway. It’s freezing cold, why does everyone have their air vents open that are blasting ice-cold air? I wrap into my blanket and hope the others are going to get cold as well. (Logbook N, intercontinental flight, September 2015)

The author of the logbook had missed the take-off of the previous flight because she had fallen asleep. The entry suggests that her activities are also meant to prevent her from falling asleep again. Clearly, she reports on the difficulties of not feeling cold, since she cannot control the other passengers’ activities. In addition to the blanket many airlines provide on long-hauls, she has brought various items with her that she uses to prevent herself from getting cold and from getting bored. These items also serve to declare (and defend) “territories of the self” (Goffman 1971: 51–86) that are potentially contested in the narrow space of the plane’s seat rows. Thus, she materially accommodates herself to the specific situation on board, using different “plane items” as a kind of “adapter” to fit her own body into the vehicle that is going to fly.

By accommodating, such “adapters” also facilitate the immobilization of bodies, which results from the commitment to remain seated. The following excerpt of a logbook reports on accommodating for an overnight flight:

Having reached our row, we stow our carry-on luggage above our seats, take off our shoes and store them as well, and put on thick socks. In my seat, I place my own pillow against the window and open the bag with the blanket, as well as the one in the next seat, since we’re lucky enough to have the row to ourselves. (Logbook B, intercontinental flight, September 2014)

Similar to the other excerpt, the author of this logbook has brought plane items with her. However, in her description the work of immobilizing the body seems to be even more obvious: Taking off her shoes and storing them suggests that she does not expect to stand up any time soon. Putting a pillow between her head and the window imitates a sleeping position and, at the same time, establishes a close interconnection between body and vehicle. Thus, the passenger establishes conditions for her own sitting that react to the peculiarities of sitting in a plane.

To sum up, hauling human bodies in airplanes requires a close connection of bodies and material infrastructure in order to prevent passengers’ bodies from being hurt or hurting others in case of turbulences. This connection however is not built once for the whole flight. Rather, it is an ongoing accomplishment (requiring certain material and social efforts) by different participants throughout the entire ride. The following section focuses on such activities.

### Incorporated into the Plane: Adapting as an Ongoing Practice

Accommodating into the flying vehicle is neither easy nor does it come without problems. Traveling in general, and in planes in particular, requires considering specific needs of human bodies, or, as Fuller puts it:

In order to travel, in order to deterritorialize, to move in a way that a human body normally couldn’t, one must first become the most basic of bodies—a body with organs—a body that runs on metabolic time. This body needs sleep, food, air and a modicum of physical activity to keep the blood pumping. (Fuller 2009: 70)

Airlines consider these needs of a basic body in the infrastructure they provide. Seats are built in a way that allows different activities like sitting, eating and (albeit inconveniently) sleeping. Oxygen supply and lighting in the cabin are regulated, flight attendants serve food and drinks, and within the narrow aisles there is some space to move. Also, many airlines provide music and movies as entertainment on medium- and long-haul flights. Yet, this infrastructure for basic needs is not perfectly adapted for anyone. Rather, passengers have to adjust their needs to the infrastructure by accommodating to the given setting. This is not done once for the whole flight, but forms an *ongoing* accomplishment, with a certain potential of failing at any time. The following logbook entry gives an impression of the ongoing work of accommodating:

It’s kind of difficult to get situated in the narrow seat: I’m still cold, so I wrap into the blanket. But I still have a beverage and snacks on the tray table in front of me, so I can barely manage to get the blanket draped around my legs. It is also difficult to reach my backpack under the seat in front of me to get to my headphones for the movie while I have all this stuff set up. But somehow, I make it work. (Logbook N, intercontinental flight, September 2015)

Since passengers are not engaged in piloting the plane, they can use the time on board for different activities.^8^ However, the range of these activities is restricted to those one can accomplish while sitting. Airplane seats are designed to be used for a variety of activities: reading, listening to music, watching movies, eating, and sleeping. As a “compromise design,” however, they do not cater equally to every need.^9^ Eating, for instance, might appear easier than sleeping. And, as the excerpt reports, these activities can hinder each other—as long as the tray table is not cleared, the passenger is even more immobile. With the opportunity to eat comes a particular restriction of moving. In addition to these material restrictions, there are rules of timing certain activities:

I’m incredibly tired and annoyed that I’m not allowed to recline my seat until we’re in the air. I get out my sleeping equipment, inflate the neck pillow. After what feels like an eternity, we take off, and as soon as we’re at altitude I recline my seat, put on my sleeping mask, and sink into a comatose sleep from which I only wake three hours later. Everything hurts and I’m terribly uncomfortable. (Logbook N, intercontinental flight, September 2015)

In this excerpt from a logbook, different time demands seem to be in conflict. The body’s (metabolic) time does not coincide with the airplane’s safety procedures. To be part of the airplane assemblage, however, demands that passengers subject their bodies’ needs to the vehicle’s rules. In Star’s (2004) terms, we find “cooperation without consent” here, accomplished in a sophisticated interaction between passengers, flight attendants, material infrastructure, and explicit rules. In a way, this is continued when the passenger finally sleeps. Although the infrastructure of the plane provides a seat that allows for sleep, this way of sleeping considerably differs from western habits of slumber (see e.g., Crook 2008; Elias 2000). Neither are we used to sleeping in a seat nor in the middle of a crowd—even though seat rows do provide passengers with a bit of privacy. Here, again, the body has to adjust to the given infrastructure. Available “adapters” like an inflatable cushion or a sleep mask do not always suffice to avoid pain as a result of the sleeping (and sitting) conditions.

Until now, I have only referred to activities (and necessities) of adapting to the conditions of a mostly quiet flight. Seen from this perspective, the airplane appears as a form of “container space” (Bissell 2007: 282) that—albeit in constant motion—harbors and encloses the passengers, their bodies and objects, thereby imposing its peculiar materialities on them. Thus, passengers mainly feel the vehicle and its materialities. However, at particular points of the flight, their horizon of feeling opens up. In regard of cars, Sheller (2004: 228), concisely states: “we not only feel the car, but we feel through the car and with the car”. In the case of air travel, this specifically applies during take-off and landing, and within turbulences. The following excerpt reports on a case of severe turbulences:

We take off. It is a very small plane with two seats on either side of the aisle: it is louder than usual, whistling and rattling. During the ascent, we fly through a large cloud and there is turbulence: It keeps feeling as if we are falling into some kind of air pocket, the plane is thrown back and forth. I hold on to the armrests. I find some amusement and distraction in the fact that some of the children on board are cheering at every bump. “Whee!” – and they are laughing and having fun. It must be like a roller coaster ride for them. Once we’re out of the cloud, the turbulences cease, and I am relieved. (Logbook U, short-haul, December 2017)

Although turbulences are at the core of safety measures, many passengers seem to add further corporeal measures to them. The author of this logbook reports that she holds on to the armrest, as if the seatbelt did not suffice to secure her body against the massive motion of the plane. In a way, thus she further intensifies the yet close connection of her body with the vehicle. At points, her report switches from an inner account about a “container space” to a quasi-external description of the plane’s course. She seems to feel “through the plane and with the plane” as it falls into air pockets. Thus, the plane seems to become a kind of extension of her body, yet—different to driving or piloting—this mainly exacerbates her lack of control.

In addition, such events often transform the atmosphere on board. Particularly, they become a shared focus for most of the passengers, which, in this excerpt is somehow materialized by the children’s cheering. Thus, the “polyfocal situation” (Schindler 2011: 128) of passengers, whose sight is limited by the narrow seat rows, changes all of a sudden into a sort of “focused interaction” (Goffman 1961: 7f.). Fellow travelers become a group with a shared fate.

In such situations, a shared focus can emerge. Usually however, passengers accomplish a polyfocal gathering instead. In this form, the presence of a whole crowd of fellow travelers and their bodies is often experienced as an important challenge of traveling (e.g., Allert 2008). In what follows, I will focus on the plane as a means of mass transport, which also characterizes the conditions of traveling it provides.

### Among the Crowd

Airliners are a medium of mass transport *par excellence*, at least in economy class. Different to most of our everyday life experiences, airliners often transport some hundred passengers. Within this crowd, different interests emerge. Some prefer cold air; others eat things they have brought with them or bring along loudly crying children. Although the atmosphere is mostly quiet and friendly, there is a potential for conflicts among passengers. Often, these conflicts are rather covertly than overtly accomplished, as a frequent flyer in one of my interviews reported:

And the thing is, you sit next to each other and there are armrests on the outside, but you have to share one in the middle. So, when I’m sitting next to someone, it’s often the case that this is some kind of conflict, fight, or negotiation that isn’t verbal. Instead it’s somehow physically, especially with men. I see it like this: okay, we’re both men. Masculinity, dominance… we both want to put our arm on the armrest now, even if we don’t really need to, and make it clear that this is my territory, I’m taking this, just because I can, because I’m manlier than you. (Interview with a frequent flyer, March 2015)

Here, the material infrastructure provides an opportunity for overt and covert conflicts that revolve around questions of social distinction between men, and—needless to mention—against women and men who might not be dominant. Accomplishing this conflict (overtly or covertly) however, requires basic cooperation between the opponents. Messmer (2003) has elaborated in detail that dispute can only be accomplished cooperatively since both sides have to keep disputing. Otherwise, the conflict closes (e.g., in favor of accomplishing a misunderstanding instead). In this excerpt, the conflict stays somehow covert, but can become overt at any point. The armrest thus provides not only an infrastructure for comfort but also an infrastructure for accomplishing conflicts that arise around territories of the self, here particularly around body-boundaries, which usually are maintained by a certain distance between different bodies to inhibit (even points of) physical contact.

The armrest may be a popular topic regarding airplane conflicts, but it is not the only one. Rather, there seem to be plenty of opportunities. In the next excerpt, “plane items” serve one passenger to accommodate to the setting, but at the same time, become a source for severe disgust for the (reporting) fellow traveler:

He sits down next to me and gets out a tablet he swipes around on. Then he opens a bag of chips. (…) He starts stuffing his face with chips, holding the tablet in his other hand. And swiping. The chewing noise is driving me insane. Also, this way of mindlessly shoving something into yourself. Disgusting. I find it inconsiderate. And I’m stuck being this close to him. And I want to tell him he’s super disgusting, but I can’t. So I turn to A. and say that I consider passenger shaming a completely legitimate practice. “Why?” I lean back to reveal the sight. “Because the acoustic space cannot be divided”. We have this thing about passenger shaming. On social media, there are pages where people are shamed for being barefoot on a plane, or doing their nails, and so on. That’s where this guy belongs. (Logbook R, short-haul, June 2016)

Here, differences in appropriate manners of eating become not only the focus of a covert conflict (and personal disgust) but also an opportunity for explicitly accomplishing conjugal agreement. With only a few insinuations that refer to an implicit but shared background (explicated to the ethnographer for whom the logbook is written), the spouses communicate their shared opinion to each other. In addition, the reference to “passenger shaming” on social media delineates such conflicts as a frequent event among air passengers, which is not reduced to momentary annoyance, but would be worth reporting to a broader audience. However, the author of the logbook does not only blame the fellow traveler but also mentions a material source for such problems: their close positions and the shared acoustics within a container space. A similar analysis can be found in the following excerpt from an interview with another frequent flyer. She told me:

What often bothers me are the smells of fellow travelers. Ugh, one time there was this old lady. She was wearing this really strong musk perfume, it was terrible. To me, it feels like the senses are on a whole different level. And odors are the worst part. They can be really obnoxious. Or, I don’t know, maybe someone buys a perfume and tries it on. It hasn’t happened to me, but that would be the worst-case scenario. (…) And the battle of the armrest. When you’re next to someone who takes up a lot of space, it can be really unpleasant. Especially on a long-haul, you’re completely helpless. (Interview with a frequent flyer, June 2016)

In this excerpt, the embodied presence of fellow travelers, in the form of wearing perfume or taking too much space, becomes a source of annoyance. However, particularly in the last sentence, the interviewee explicitly draws a connection between her own sensations and the vehicle. Her impression that her senses are different on board seems to emphasize and to extend Fuller’s aforementioned concept that flying requires becoming a “basic body”. It emphasizes the concept since the body seems to be more presently felt than in everyday life, and it extends it because the body’s sensations seem to change or at least intensify in the vehicle. Also, it suggests that air traveling bodies are not reduced to basic ones. Rather, they appear as heavily socialized bodies with perfume, clothing, senses, (differing) habits, discipline, and so forth. These (potentially) challenge the integrity of body-boundaries inter alia by jeopardizing acoustic or olfactory spaces.

To conclude, the atmosphere on an aircraft is heavily characterized by the socio-technical density of the vehicle. Not only do passengers actively accommodate to the infrastructure of the airplane, they also have to find ways to cooperate (with or without consent) with the other passengers on board who come along with their bodies (and personal items) that smell, make noises, and demand territory. Numerous studies have shown that a large part of flight attendants’ work consists of establishing a friendly atmosphere on board (e.g., Hochschild 2012; Lin 2015). Indeed, (most) passengers and their bodies cooperate in this endeavor by concertedly accomplishing an atmosphere of “civil inattention” in a crowded and dense space. However, as this section has shown, this involves certain challenges. It requires an active adaptation to the material infrastructure and the social circumstances as well as constant attention to body-boundaries and territories of the self. Passengering is not least accomplished by “doing being ordinary” (Sacks 1984) in a (materially and therefor also socially) extra-ordinary (and in many ways tiring) setting.

### Detaching

Within the airplane assemblage, bodies and objects are materially interconnected in order to accomplish a safe flight, requiring that they continuously adapt to the infrastructure of the plane. However, it is only assembled for the timespan of a flight. It has to be detached when the flight ends. In this process, the individuals loosen their connection with the vehicle in order to re-build their own vehicular unit. In what follows, I will focus on this process of detaching, distinguishing two different forms: firstly, we find a temporary detaching when passengers get up from their seats and move as another vehicular unit within the plane; secondly, there is the ultimate form of detaching in order to leave the plane and finish the flight.

The first, temporary form of building a separate vehicular unit within the plane occurs only seldomly since the infrastructure of airplanes impedes it whenever possible. Instead, as I have elaborated above, passengers’ bodies are bound to their seats by the material design as well as by explicit instructions, while flight attendants move through the plane in order to provide passengers with food and drinks as well as taking away leftovers. Only for those activities that cannot be handed over to flight attendants or material infrastructure, passengers get up from their seats to move within the plane. Mainly, they go to use restrooms or to accomplish little movements as exercise against embolism. However, this movement encounters different difficulties as the following excerpt from my field notes reports:

While eating, I notice that I need to go to the restrooms. However, I don’t want to disturb my seat neighbor, and thus I decide to wait. Anyway, it would be difficult to get there—as long as flight attendants are still walking with their trolleys, it is difficult to pass. (Field notes of a short-haul flight, October 2013)

In this excerpt, various difficulties of (relatively) independent movement within an infrastructure of sitting are mentioned. Getting up is often inconvenient to seat neighbors, since the seat rows are narrow. The equally narrow aisle only leaves room for one person. Detaching is just as involved as accommodating since the vehicle’s setting doesn’t release its parts easily.

At the end of a flight however, many people seem to actively seek to detach from the plane assemblage by getting up from their seats as soon as possible. The following excerpt from a frequent traveler’s logbook reports on getting off the plane in detail:

Shortly after, there is an announcement that seats are to be put in the upright position and window blinds are to be opened. All electronic devices must be deactivated and the seatbelt sign is switched on, and we are reminded of it once again. The captain says a few things about the weather and the landing approach. Should be no problem, visibility is good and it’s not raining. The flaps are extended and I can feel the landing gear extend. Somehow, one focuses more on the landing than on the take-off. You can really feel the descent and are busy swallowing to help adjust to the pressure. The plane accelerates one last time, and you touch down. After that, it’s always the same scene. Announcement: Please remain seated until the seatbelt sign is switched off and the plane stops moving at the gate. It’s a good thing this announcement exists, but it is futile. As soon as the plane comes to a stop, most people jump out of their seats like maniacs and get their belongings from the overhead bins, standing in the aisles and blocking everything. You could almost think there’s a fire or you’ll be denied entry at the border if you’re not fast enough, even though it will be a while before everyone can get off the plane. (Logbook B, intercontinental flight, September 2014)

Similar to conversations, closings do not just happen in air travel. Complex activities of “opening up closings” (Schegloff and Sacks 1973) take place in airplanes as well. Often, there are formalized rituals like the landing announcement, as the logbook mentions, that emphasize possible dangers of a flight. Requesting to move the seat back into the upright position, to open window blinds, or to switch off electronic devices also indicate possible dangers. At the same time, passengers are reminded to contribute to the security of landing by suspending their activities and come back to the position of take-off. This announcement at the end of the flight, right before thanking passengers to have chosen the airline for their travel, requests one last intensification of the interconnection of bodies and vehicle.

Reminded of their embodied existence (and their vulnerability to injuries), passengers are once more immobilized since even the few possibilities for small movements within the space of their seats are withdrawn again. Bound to the vehicle, the boundaries of one’s body in a way extend into the plane. Some passengers report on deeply corporeal sensations like altitude compensation (in the excerpt above), the embodied sensation of losing height and landing, or particular fears. While landing, maybe particularly during touch-down, bodies that were bound to their seats receive a physical impulse. The rationality of getting up early is certainly questionable, as the logbook suggests. However, it might be a consequence of this physical impulse. Also, by getting one’s hand luggage from the overhead bins and re-connecting mobile phones, the individual prepares for detaching from the plane and becoming a vehicular unit of its own again.

Thus, at the end of the ride, the interconnection of bodies and objects has to be released bit by bit. The urgency put forward by many passengers at this point again illustrates the difficulties and complexities of adapting to the vehicle’s infrastructure. These difficulties include the paradox of giving up one’s own mobility in order to be incorporated into the vehicle’s mobility, which is associated with further losses of autonomy.

## Conclusion

The material interrelations between bodies and objects are a wide, worthwhile, and absorbing field, which has not sufficiently been researched yet. Focusing on the interconnections of bodies and objects within air travel, this article contributes to its exploration. It delineates that such an interconnection does not just happen, but has to be accomplished continuously by different participants with a certain risk of failure at many points. Within mobilities, such processes of connecting occur under the specific circumstances of a (most of the time) moving vehicle, which involves vibrations and sometimes also bumpy travel segments. In addition, one is bound to the vehicle as long as it is in motion.

Viewed from this perspective, sociality on board of an aircraft is a complex social process which depends on different (material) participants like the crew, passengers, their bodies, objects, and material infrastructure. Not only flying but also boarding is clearly shaped by the materiality of the vehicle that is technically designed to leave the ground. Passengers coming in have to practically (and accountably) transform from being a vehicular unit of their own to becoming part of a hetero-mobile by fitting their bodies into the vehicle’s narrow seat rows and immobilizing them, while flight attendants move through the plane in their stead. To fit into this “infrastructure of sitting,” passengers use different objects as “adapters” to accommodate to the given setting. While accomplishing this deeply material interconnection, they mostly contribute to a culture of “civil inattention” among the passengers. Even conflict is more often covertly than overtly accomplished. However, the same infrastructure that contributes to the mostly friendly and peaceful atmosphere on board can turn into an infrastructure for conflicts: seat rests, arm rests, food, drinks, bodies of other passengers and their smells or noises, they all can be annoying, since they challenge the integrity of body-boundaries. At the end of the ride, the whole airplane assemblage has to detach again. Therefore, passengers loosen their connection with the vehicle bit by bit and finally come to move as a vehicular unit of their own again. From being air passengers (in a narrow sense), they return to being travelers by reassembling a unit that is able to move out of the plane, through the airport, and to the next vehicle, be it a connecting flight or another means of transport.

From a theoretical standpoint, these findings suggest further investigating the continuous modification that material assemblages built of persons, their bodies and objects undergo even when “only” staying immobile during a flight. Passengers do not just enter and leave a vehicle staying identical with themselves. Rather, they are continuously adapting to a material infrastructure, which shapes and adjusts their corporeal needs and capacities, including their very senses. The dynamic assemblages of bodies and objects are key to these processes of adaptation.

## Footnotes

1 This research project was financially supported by the German Research Foundation (Deutsche Forschungsgemeinschaft) under grant number 271437443.

2 A detailed discussion of logbooks can be found elsewhere (Schindler 2020a).

3 As Hirschauer (2004) rightly criticizes, the otherwise very convincing analytical input of actor-network theories does not provide proper vocabulary for conceptualizing the sociality of (human) bodies. Having this critique in mind, my use of the concept “assemblage” follows Dant (2004), who incorporated this dimension by a phenomenological extension of the concept.

4 Although perhaps underestimated, waiting is a common phenomenon of modern coordination and contemporary lives. In the last few years, it has gained a growing analytical attention (e.g., Ayaß 2020; Göttlich 2016), also in regard of (aero)mobilities (e.g., Belvedere and López 2016; Bissell 2007). Within air travel, waiting is not only frequent but at many points actually part of the practice. I have discussed this phenomenon in detail elsewhere (Schindler 2020b).

5 I would not have reported similarly about “extraordinary ease,” had I gone by train. On the contrary, I would have marked the first excerpt as an extraordinary difficult situation.

6 See Adey (2006) for an impressive analysis of the relativity of mobility and immobility.

7 See Huff (2009) for discourses on discrimination against overweight people on airplanes.

8 A detailed discussion of different (sometimes conflicting) temporalities on board can be found elsewhere (Schindler 2020b).

9 Here, I borrow from Potthast (2016: 143f.), who characterizes airports as “architectures of compromise”.
